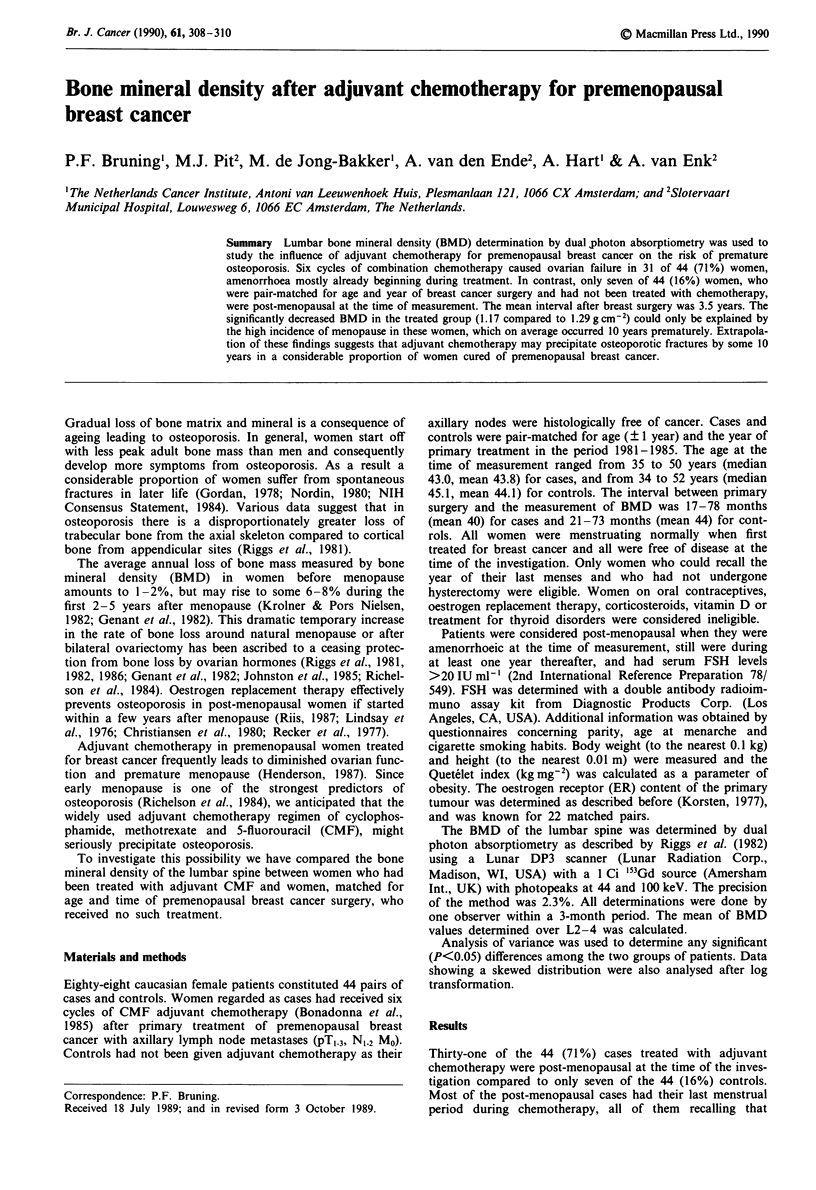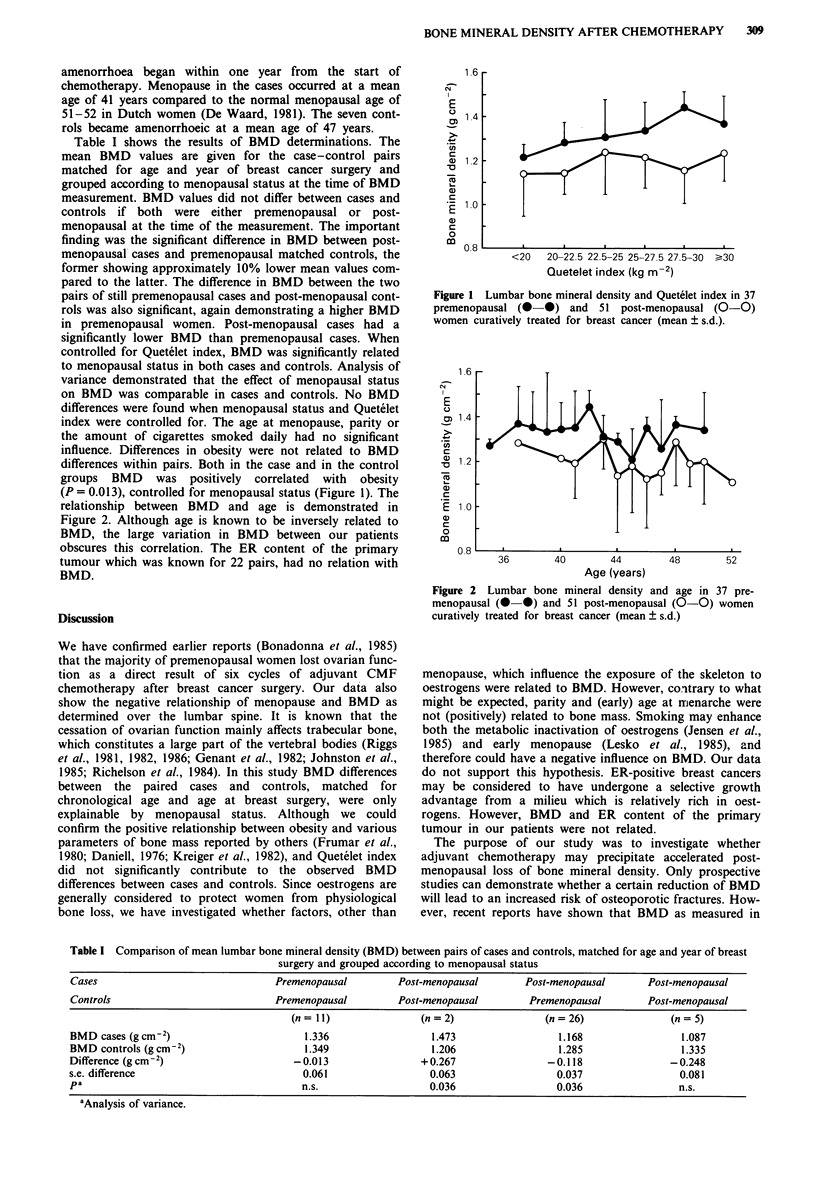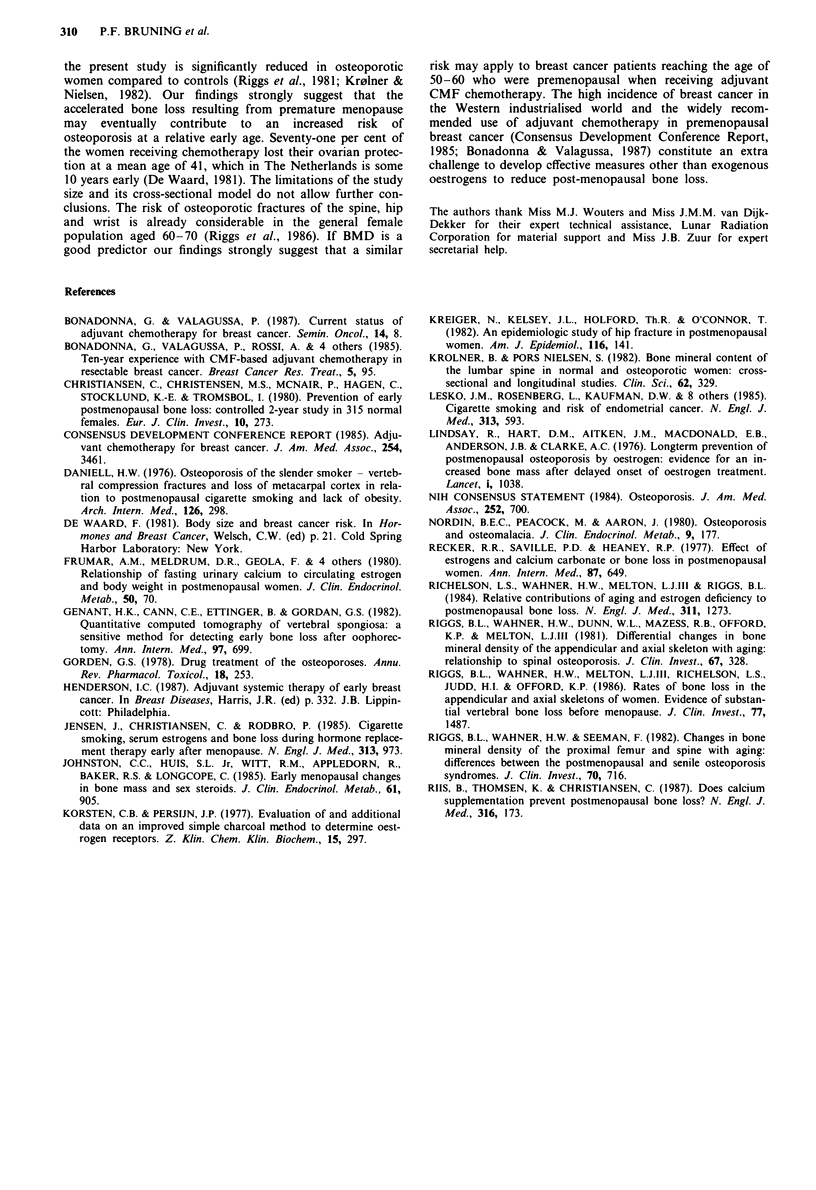# Bone mineral density after adjuvant chemotherapy for premenopausal breast cancer.

**DOI:** 10.1038/bjc.1990.58

**Published:** 1990-02

**Authors:** P. F. Bruning, M. J. Pit, M. de Jong-Bakker, A. van den Ende, A. Hart, A. van Enk

**Affiliations:** The Netherlands Cancer Institute, Antoni van Leeuwenhoek Huis, Amsterdam.

## Abstract

Lumbar bone mineral density (BMD) determination by dual photon absorptiometry was used to study the influence of adjuvant chemotherapy for premenopausal breast cancer on the risk of premature osteoporosis. Six cycles of combination chemotherapy caused ovarian failure in 31 of 44 (71%) women, amenorrhoea mostly already beginning during treatment. In contrast, only seven of 44 (16%) women, who were pair-matched for age and year of breast cancer surgery and had not been treated with chemotherapy, were post-menopausal at the time of measurement. The mean interval after breast surgery was 3.5 years. The significantly decreased BMD in the treated group (1.17 compared to 1.29 g cm-2) could only be explained by the high incidence of menopause in these women, which on average occurred 10 years prematurely. Extrapolation of these findings suggests that adjuvant chemotherapy may precipitate osteoporotic fractures by some 10 years in a considerable proportion of women cured of premenopausal breast cancer.


Br. J. Cancer (1990), 61, 308–310 © Macmillan Press Ltd., 1990

### Bone mineral density after adjuvant chemotherapy for premenopausal breast cancer

P.F. Bruning[1], M.J. Pit[2], M. de Jong-Bakker[1], A. van den Ende[2], A. Hart[1] & A. van Enk[2]

[1]*The Netherlands Cancer Institute, Antoni van Leeuwenhoek Huis, Plesmanlaan 121, 1066 CX Amsterdam; and* [2]*Slotervaart Municipal Hospital, Louwesweg 6, 1066 EC Amsterdam, The Netherlands.*

**Summary** Lumbar bone mineral density (BMD) determination by dual photon absorptiometry was used to study the influence of adjuvant chemotherapy for premenopausal breast cancer on the risk of premature osteoporosis. Six cycles of combination chemotherapy caused ovarian failure in 31 of 44 (71%) women, amenorrhoea mostly already beginning during treatment. In contrast, only seven of 44 (16%) women, who were pair-matched for age and year of breast cancer surgery and had not been treated with chemotherapy, were post-menopausal at the time of measurement. The mean interval after breast surgery was 3.5 years. The significantly decreased BMD in the treated group (1.17 compared to 1.29 g cm$^{-2}$) could only be explained by the high incidence of menopause in these women, which on average occurred 10 years prematurely. Extrapolation of these findings suggests that adjuvant chemotherapy may precipitate osteoporotic fractures by some 10 years in a considerable proportion of women cured of premenopausal breast cancer.

Gradual loss of bone matrix and mineral is a consequence of ageing leading to osteoporosis. In general, women start off with less peak adult bone mass than men and consequently develop more symptoms from osteoporosis. As a result a considerable proportion of women suffer from spontaneous fractures in later life (Gordan, 1978; Nordin, 1980; NIH Consensus Statement, 1984). Various data suggest that in osteoporosis there is a disproportionately greater loss of trabecular bone from the axial skeleton compared to cortical bone from appendicular sites (Riggs *et al.*, 1981).

The average annual loss of bone mass measured by bone mineral density (BMD) in women before menopause amounts to 1–2%, but may rise to some 6–8% during the first 2–5 years after menopause (Krolner & Pors Nielsen, 1982; Genant *et al.*, 1982). This dramatic temporary increase in the rate of bone loss around natural menopause or after bilateral ovariectomy has been ascribed to a ceasing protection from bone loss by ovarian hormones (Riggs *et al.*, 1981, 1982, 1986; Genant *et al.*, 1982; Johnston *et al.*, 1985; Richelson *et al.*, 1984). Oestrogen replacement therapy effectively prevents osteoporosis in post-menopausal women if started within a few years after menopause (Riis, 1987; Lindsay *et al.*, 1976; Christiansen *et al.*, 1980; Recker *et al.*, 1977).

Adjuvant chemotherapy in premenopausal women treated for breast cancer frequently leads to diminished ovarian function and premature menopause (Henderson, 1987). Since early menopause is one of the strongest predictors of osteoporosis (Richelson *et al.*, 1984), we anticipated that the widely used adjuvant chemotherapy regimen of cyclophosphamide, methotrexate and 5-fluorouracil (CMF), might seriously precipitate osteoporosis.

To investigate this possibility we have compared the bone mineral density of the lumbar spine between women who had been treated with adjuvant CMF and women, matched for age and time of premenopausal breast cancer surgery, who received no such treatment.

### Materials and methods

Eighty-eight caucasian female patients constituted 44 pairs of cases and controls. Women regarded as cases had received six cycles of CMF adjuvant chemotherapy (Bonadonna *et al.*, 1985) after primary treatment of premenopausal breast cancer with axillary lymph node metastases ($pT_{1-3}$, $N_{1-2}$ $M_0$). Controls had not been given adjuvant chemotherapy as their axillary nodes were histologically free of cancer. Cases and controls were pair-matched for age (±1 year) and the year of primary treatment in the period 1981–1985. The age at the time of measurement ranged from 35 to 50 years (median 43.0, mean 43.8) for cases, and from 34 to 52 years (median 45.1, mean 44.1) for controls. The interval between primary surgery and the measurement of BMD was 17–78 months (mean 40) for cases and 21–73 months (mean 44) for controls. All women were menstruating normally when first treated for breast cancer and all were free of disease at the time of the investigation. Only women who could recall the year of their last menses and who had not undergone hysterectomy were eligible. Women on oral contraceptives, oestrogen replacement therapy, corticosteroids, vitamin D or treatment for thyroid disorders were considered ineligible.

Patients were considered post-menopausal when they were amenorrhoeic at the time of measurement, still were during at least one year thereafter, and had serum FSH levels $>20$ IU ml$^{-1}$ (2nd International Reference Preparation 78/549). FSH was determined with a double antibody radioimmuno assay kit from Diagnostic Products Corp. (Los Angeles, CA, USA). Additional information was obtained by questionnaires concerning parity, age at menarche and cigarette smoking habits. Body weight (to the nearest 0.1 kg) and height (to the nearest 0.01 m) were measured and the Quetélet index (kg mg$^{-2}$) was calculated as a parameter of obesity. The oestrogen receptor (ER) content of the primary tumour was determined as described before (Korsten, 1977), and was known for 22 matched pairs.

The BMD of the lumbar spine was determined by dual photon absorptiometry as described by Riggs *et al.* (1982) using a Lunar DP3 scanner (Lunar Radiation Corp., Madison, WI, USA) with a 1 Ci $^{153}$Gd source (Amersham Int., UK) with photopeaks at 44 and 100 keV. The precision of the method was 2.3%. All determinations were done by one observer within a 3-month period. The mean of BMD values determined over L2–4 was calculated.

Analysis of variance was used to determine any significant ($P<0.05$) differences among the two groups of patients. Data showing a skewed distribution were also analysed after log transformation.

### Results

Thirty-one of the 44 (71%) cases treated with adjuvant chemotherapy were post-menopausal at the time of the investigation compared to only seven of the 44 (16%) controls. Most of the post-menopausal cases had their last menstrual period during chemotherapy, all of them recalling that

Correspondence: P.F. Bruning.
Received 18 July 1989; and in revised form 3 October 1989.

amenorrhoea began within one year from the start of chemotherapy. Menopause in the cases occurred at a mean age of 41 years compared to the normal menopausal age of 51–52 in Dutch women (De Waard, 1981). The seven controls became amenorrhoeic at a mean age of 47 years.

Table I shows the results of BMD determinations. The mean BMD values are given for the case–control pairs matched for age and year of breast cancer surgery and grouped according to menopausal status at the time of BMD measurement. BMD values did not differ between cases and controls if both were either premenopausal or post-menopausal at the time of the measurement. The important finding was the significant difference in BMD between post-menopausal cases and premenopausal matched controls, the former showing approximately 10% lower mean values compared to the latter. The difference in BMD between the two pairs of still premenopausal cases and post-menopausal controls was also significant, again demonstrating a higher BMD in premenopausal women. Post-menopausal cases had a significantly lower BMD than premenopausal cases. When controlled for Quetélet index, BMD was significantly related to menopausal status in both cases and controls. Analysis of variance demonstrated that the effect of menopausal status on BMD was comparable in cases and controls. No BMD differences were found when menopausal status and Quetélet index were controlled for. The age at menopause, parity or the amount of cigarettes smoked daily had no significant influence. Differences in obesity were not related to BMD differences within pairs. Both in the case and in the control groups BMD was positively correlated with obesity ($P = 0.013$), controlled for menopausal status (Figure 1). The relationship between BMD and age is demonstrated in Figure 2. Although age is known to be inversely related to BMD, the large variation in BMD between our patients obscures this correlation. The ER content of the primary tumour which was known for 22 pairs, had no relation with BMD.

**Figure 1** Lumbar bone mineral density and Quetélet index in 37 premenopausal (●—●) and 51 post-menopausal (O—O) women curatively treated for breast cancer (mean ± s.d.).

**Figure 2** Lumbar bone mineral density and age in 37 premenopausal (●—●) and 51 post-menopausal (O—O) women curatively treated for breast cancer (mean ± s.d.)

### Discussion

We have confirmed earlier reports (Bonadonna *et al.*, 1985) that the majority of premenopausal women lost ovarian function as a direct result of six cycles of adjuvant CMF chemotherapy after breast cancer surgery. Our data also show the negative relationship of menopause and BMD as determined over the lumbar spine. It is known that the cessation of ovarian function mainly affects trabecular bone, which constitutes a large part of the vertebral bodies (Riggs *et al.*, 1981, 1982, 1986; Genant *et al.*, 1982; Johnston *et al.*, 1985; Richelson *et al.*, 1984). In this study BMD differences between the paired cases and controls, matched for chronological age and age at breast surgery, were only explainable by menopausal status. Although we could confirm the positive relationship between obesity and various parameters of bone mass reported by others (Frumar *et al.*, 1980; Daniell, 1976; Kreiger *et al.*, 1982), and Quetélet index did not significantly contribute to the observed BMD differences between cases and controls. Since oestrogens are generally considered to protect women from physiological bone loss, we have investigated whether factors, other than menopause, which influence the exposure of the skeleton to oestrogens were related to BMD. However, contrary to what might be expected, parity and (early) age at menarche were not (positively) related to bone mass. Smoking may enhance both the metabolic inactivation of oestrogens (Jensen *et al.*, 1985) and early menopause (Lesko *et al.*, 1985), and therefore could have a negative influence on BMD. Our data do not support this hypothesis. ER-positive breast cancers may be considered to have undergone a selective growth advantage from a milieu which is relatively rich in oestrogens. However, BMD and ER content of the primary tumour in our patients were not related.

The purpose of our study was to investigate whether adjuvant chemotherapy may precipitate accelerated post-menopausal loss of bone mineral density. Only prospective studies can demonstrate whether a certain reduction of BMD will lead to an increased risk of osteoporotic fractures. However, recent reports have shown that BMD as measured in

**Table I** Comparison of mean lumbar bone mineral density (BMD) between pairs of cases and controls, matched for age and year of breast surgery and grouped according to menopausal status

| *Cases* | *Premenopausal* | *Post-menopausal* | *Post-menopausal* | *Post-menopausal* |
|---|---|---|---|---|
| *Controls* | *Premenopausal* | *Post-menopausal* | *Premenopausal* | *Post-menopausal* |
| | ($n = 11$) | ($n = 2$) | ($n = 26$) | ($n = 5$) |
| BMD cases ($g\,cm^{-2}$) | 1.336 | 1.473 | 1.168 | 1.087 |
| BMD controls ($g\,cm^{-2}$) | 1.349 | 1.206 | 1.285 | 1.335 |
| Difference ($g\,cm^{-2}$) | −0.013 | +0.267 | −0.118 | −0.248 |
| s.e. difference | 0.061 | 0.063 | 0.037 | 0.081 |
| $P^a$ | n.s. | 0.036 | 0.036 | n.s. |

[a]Analysis of variance.

the present study is significantly reduced in osteoporotic women compared to controls (Riggs *et al.*, 1981; Krølner & Nielsen, 1982). Our findings strongly suggest that the accelerated bone loss resulting from premature menopause may eventually contribute to an increased risk of osteoporosis at a relative early age. Seventy-one per cent of the women receiving chemotherapy lost their ovarian protection at a mean age of 41, which in The Netherlands is some 10 years early (De Waard, 1981). The limitations of the study size and its cross-sectional model do not allow further conclusions. The risk of osteoporotic fractures of the spine, hip and wrist is already considerable in the general female population aged 60–70 (Riggs *et al.*, 1986). If BMD is a good predictor our findings strongly suggest that a similar risk may apply to breast cancer patients reaching the age of 50–60 who were premenopausal when receiving adjuvant CMF chemotherapy. The high incidence of breast cancer in the Western industrialised world and the widely recommended use of adjuvant chemotherapy in premenopausal breast cancer (Consensus Development Conference Report, 1985; Bonadonna & Valagussa, 1987) constitute an extra challenge to develop effective measures other than exogenous oestrogens to reduce post-menopausal bone loss.

The authors thank Miss M.J. Wouters and Miss J.M.M. van Dijk-Dekker for their expert technical assistance, Lunar Radiation Corporation for material support and Miss J.B. Zuur for expert secretarial help.

### References

BONADONNA, G. & VALAGUSSA, P. (1987). Current status of adjuvant chemotherapy for breast cancer. *Semin. Oncol.*, **14**, 8.

BONADONNA, G., VALAGUSSA, P., ROSSI, A. & 4 others (1985). Ten-year experience with CMF-based adjuvant chemotherapy in resectable breast cancer. *Breast Cancer Res. Treat.*, **5**, 95.

CHRISTIANSEN, C., CHRISTENSEN, M.S., MCNAIR, P., HAGEN, C., STOCKLUND, K.-E. & TROMSBOL, I. (1980). Prevention of early postmenopausal bone loss: controlled 2-year study in 315 normal females. *Eur. J. Clin. Invest.*, **10**, 273.

CONSENSUS DEVELOPMENT CONFERENCE REPORT (1985). Adjuvant chemotherapy for breast cancer. *J. Am. Med. Assoc.*, **254**, 3461.

DANIELL, H.W. (1976). Osteoporosis of the slender smoker – vertebral compression fractures and loss of metacarpal cortex in relation to postmenopausal cigarette smoking and lack of obesity. *Arch. Intern. Med.*, **126**, 298.

DE WAARD, F. (1981). Body size and breast cancer risk. In *Hormones and Breast Cancer*, Welsch, C.W. (ed) p. 21. Cold Spring Harbor Laboratory: New York.

FRUMAR, A.M., MELDRUM, D.R., GEOLA, F. & 4 others (1980). Relationship of fasting urinary calcium to circulating estrogen and body weight in postmenopausal women. *J. Clin. Endocrinol. Metab.*, **50**, 70.

GENANT, H.K., CANN, C.E., ETTINGER, B. & GORDAN, G.S. (1982). Quantitative computed tomography of vertebral spongiosa: a sensitive method for detecting early bone loss after oophorectomy. *Ann. Intern. Med.*, **97**, 699.

GORDEN, G.S. (1978). Drug treatment of the osteoporoses. *Annu. Rev. Pharmacol. Toxicol.*, **18**, 253.

HENDERSON, I.C. (1987). Adjuvant systemic therapy of early breast cancer. In *Breast Diseases*, Harris, J.R. (ed) p. 332. J.B. Lippincott: Philadelphia.

JENSEN, J., CHRISTIANSEN, C. & RODBRO, P. (1985). Cigarette smoking, serum estrogens and bone loss during hormone replacement therapy early after menopause. *N. Engl. J. Med.*, **313**, 973.

JOHNSTON, C.C., HUIS, S.L. Jr, WITT, R.M., APPLEDORN, R., BAKER, R.S. & LONGCOPE, C. (1985). Early menopausal changes in bone mass and sex steroids. *J. Clin. Endocrinol. Metab.*, **61**, 905.

KORSTEN, C.B. & PERSIJN, J.P. (1977). Evaluation of and additional data on an improved simple charcoal method to determine oestrogen receptors. *Z. Klin. Chem. Klin. Biochem.*, **15**, 297.

KREIGER, N., KELSEY, J.L., HOLFORD, Th.R. & O'CONNOR, T. (1982). An epidemiologic study of hip fracture in postmenopausal women. *Am. J. Epidemiol.*, **116**, 141.

KROLNER, B. & PORS NIELSEN, S. (1982). Bone mineral content of the lumbar spine in normal and osteoporotic women: cross-sectional and longitudinal studies. *Clin. Sci.*, **62**, 329.

LESKO, J.M., ROSENBERG, L., KAUFMAN, D.W. & 8 others (1985). Cigarette smoking and risk of endometrial cancer. *N. Engl. J. Med.*, **313**, 593.

LINDSAY, R., HART, D.M., AITKEN, J.M., MACDONALD, E.B., ANDERSON, J.B. & CLARKE, A.C. (1976). Longterm prevention of postmenopausal osteoporosis by oestrogen: evidence for an increased bone mass after delayed onset of oestrogen treatment. *Lancet*, **i**, 1038.

NIH CONSENSUS STATEMENT (1984). Osteoporosis. *J. Am. Med. Assoc.*, **252**, 700.

NORDIN, B.E.C., PEACOCK, M. & AARON, J. (1980). Osteoporosis and osteomalacia. *J. Clin. Endocrinol. Metab.*, **9**, 177.

RECKER, R.R., SAVILLE, P.D. & HEANEY, R.P. (1977). Effect of estrogens and calcium carbonate or bone loss in postmenopausal women. *Ann. Intern. Med.*, **87**, 649.

RICHELSON, L.S., WAHNER, H.W., MELTON, L.J.III & RIGGS, B.L. (1984). Relative contributions of aging and estrogen deficiency to postmenopausal bone loss. *N. Engl. J. Med.*, **311**, 1273.

RIGGS, B.L., WAHNER, H.W., DUNN, W.L., MAZESS, R.B., OFFORD, K.P. & MELTON, L.J.III (1981). Differential changes in bone mineral density of the appendicular and axial skeleton with aging: relationship to spinal osteoporosis. *J. Clin. Invest.*, **67**, 328.

RIGGS, B.L., WAHNER, H.W., MELTON, L.J.III, RICHELSON, L.S., JUDD, H.I. & OFFORD, K.P. (1986). Rates of bone loss in the appendicular and axial skeletons of women. Evidence of substantial vertebral bone loss before menopause. *J. Clin. Invest.*, **77**, 1487.

RIGGS, B.L., WAHNER, H.W. & SEEMAN, F. (1982). Changes in bone mineral density of the proximal femur and spine with aging: differences between the postmenopausal and senile osteoporosis syndromes. *J. Clin. Invest.*, **70**, 716.

RIIS, B., THOMSEN, K. & CHRISTIANSEN, C. (1987). Does calcium supplementation prevent postmenopausal bone loss? *N. Engl. J. Med.*, **316**, 173.